# The Texture and Structure of the Melt-Spun Co_2_MnAl-Type Heusler Alloy

**DOI:** 10.3390/ma14030501

**Published:** 2021-01-21

**Authors:** Pavel Diko, Viktor Kavečanský, Tomáš Ryba, Lucia Frolová, Rastislav Varga, Zuzana Vargová

**Affiliations:** 1 Institute of Experimental Physics, Slovak Academy of Sciences, 04001 Košice, Slovakia; viktor.kavecansky@saske.sk; 2 Institute of Physics, Pavol Jozef Šafárik University in Košice, 04001 Košice, Slovakia; ryba@rvmagnetics.com (T.R.); lucia.frolova@upjs.sk (L.F.); rastislav.varga@upjs.sk (R.V.); 3 Institute of Chemistry, Pavol Jozef Šafárik University in Košice, 04154 Košice, Slovakia; zuzana.vargova@upjs.sk

**Keywords:** Co_2_MnAl Heusler alloy, solidification, microstructure, partitionless growth, texture

## Abstract

The structure of the Co_2_MnAl_-_type Heusler alloy in the form of a melt-spun ribbon was studied by electron microscopy, electron back-scattered diffraction (EBSD), and X-ray diffraction. The melt-spun ribbon consists of a homogeneous single-phase disordered Heusler alloy at the wheel side of the ribbon and an inhomogeneous single-phase alloy, formed by cellular or dendritic growth, at the free surface of the ribbon. Cellular growth causes the formation of an inhomogeneous distribution of the elemental constituents, with a higher Co and Al concentration in the centre of the cells or dendritic arms and a higher concentration of Mn at the cell boundaries. The EBSD analysis shows that the columnar crystals grow in the <111> crystal direction and are declined by about 10° against the direction of the spinning.

## 1. Introduction

Co_2_MnAl-type Heusler alloys are studied for their high magnetic moment and relatively high spin polarization [1,2,3,4]. Generally, Heusler alloys seem to be the material of choice for many applications due to their tunable electronic structures, which allow for the design of desirable properties. Heusler alloys exist in a wide concentration range and their properties are significantly influenced by their chemical compositions. They are usually prepared by arc-melting. After slow cooling, the cast samples have a multiphase structure (occurrence of peritectic reaction [5]); therefore, annealing is necessary for sample homogenization [6,7]. More recently, a new method for the preparation of Heusler alloys has been introduced which uses rapid quenching by melt spinning, providing very fast cooling rates of 10^5^–10^6^ K/s [8,9,10,11,12,13]. This may offer two desirable advantages: the avoidance of thermal annealing to reach a homogeneous single-phase alloy and the production of highly textured polycrystalline ribbons with improved properties along a specific direction. However, information on the solidification mechanism, microstructure formation, and crystal alignment (texture) is not satisfactory, nor are the microscopic homogeneities of the melt-spun ribbons’ chemical compositions. For example, electron back-scattering diffraction analysis, used for the detailed study of grain size, shape, and crystal alignment [14,15], has not yet been performed on melt-spun Heusler alloys. In this paper, we study the structure and source of the compositional inhomogeneity in Co_2_MnAl-type Heusler alloys prepared by melt spinning.

## 2. Materials and Methods

As-cast pellets of nominal composition—Co_2_MnAl—were prepared by Ar arc-melting from pure elements (>99.9%) in an Edmund–Buhler MAM1 arc-melter (EdmundBuhler GmbH, Bodelshausen, Germany). Ribbon pieces about 1 mm wide, 40 μm thick, and 5 cm long were produced by melt spinning (the ribbon broke into pieces during spinning) in a helium atmosphere at a wheel linear speed of 20 ms^−1^. It was shown using an EDS microanalyser (Oxford Instruments, High Wycombe, UK) that the chemical composition of the obtained alloy was Co_2.00_Mn_1.28_Al_1.06_ (in wt. %: 54.37 Co, 32.44 Mn, 13.9 Al) with its stoichiometry shifted to Mn, apparently due to changes in composition during arc-melting. The microstructure of the samples was studied by a scanning electron microscope (SEM) (Tescan, Brno–Kohoutovice, Czech Republic) in the secondary electron (SE) or back-scattered electron (BSE) regimes equipped with an energy dispersive X-ray analyser (EDS) and an electron back-scattered diffraction (EBSD) analyser (Oxford Instruments, High Wycombe, UK). A two-dimensional diffraction pattern of the Co_2_MnAl melt-spun ribbon was taken in the transmission geometry using a Rigaku D/MAX Rapid II X-ray diffractometer (Rigaku Corporation, Tokyo, Japan) with an image-plate detector, Mo K_α_ radiation (50 kV, 38 mA), and a collimator diameter of 100 μm. The measured pattern was consequently converted into one-dimensional intensity vs. diffraction angle dependence (Rigaku 2DP software) and processed by standard procedures for the qualitative phase analysis and the Rietveld crystal structure refinement method.

## 3. Results

**Figure 1:**
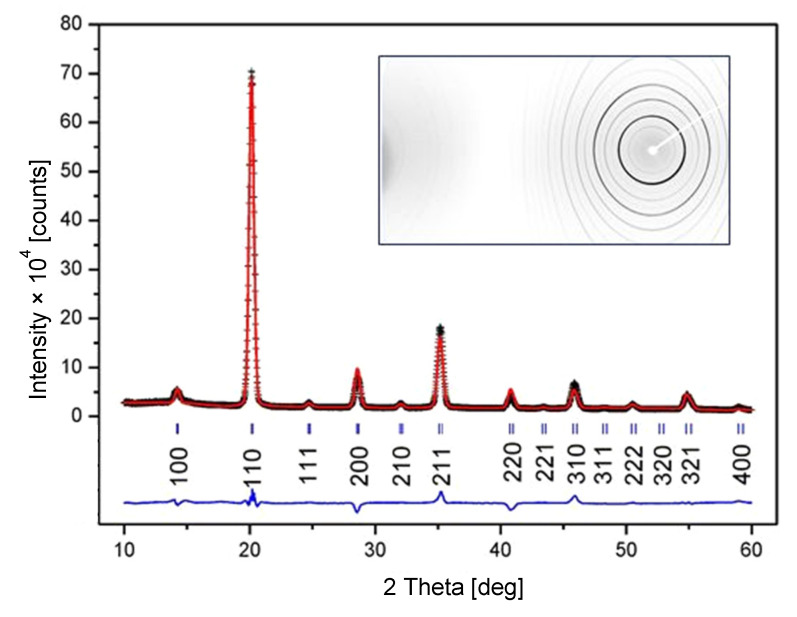
Two-dimensional X-ray diffraction pattern for Co_2_MnAl ribbon (transmission geometry), with the measured pattern consequently converted into a one-dimensional intensity vs. diffraction angle dependence and processed by standard procedures for qualitative phase analysis and the Rietveld crystal structure refinement method. The phase composition of the sample has been found to be single phase AlCo_2_Mn (the *Fm3-m* space group, JCPDS card No: 03-065-5185).

According to the X-ray analysis, the composition of the sample is a single-phase Co_2_AlMn with the lattice parameter *a* = 5.671(8) Å. The two-dimensional diffractogram indicates randomly oriented fine crystals without preferential orientation. However, if the measured diffraction pattern of Co_2_AlMn is indexed in the *Fm3-m* space group (Figure 1), then the intensities of the fcc-typical (111), (311), and (331) diffractions are negligible compared to those of other peaks. This may mean that, at least in some parts of the sample, the crystal structure is disordered. This disorder can most probably be explained by the formation of the B2 (CsCl-like) type of crystal structure, with the equivalent occupation of Al and Mn positions leading to reduced symmetry (space group *Pm3-m*). The diffraction pattern can be indexed using a primitive unit cell, with the lattice constant being half the lattice parameter of the prototype *Fm3-m* structure.

**Figure 2:**
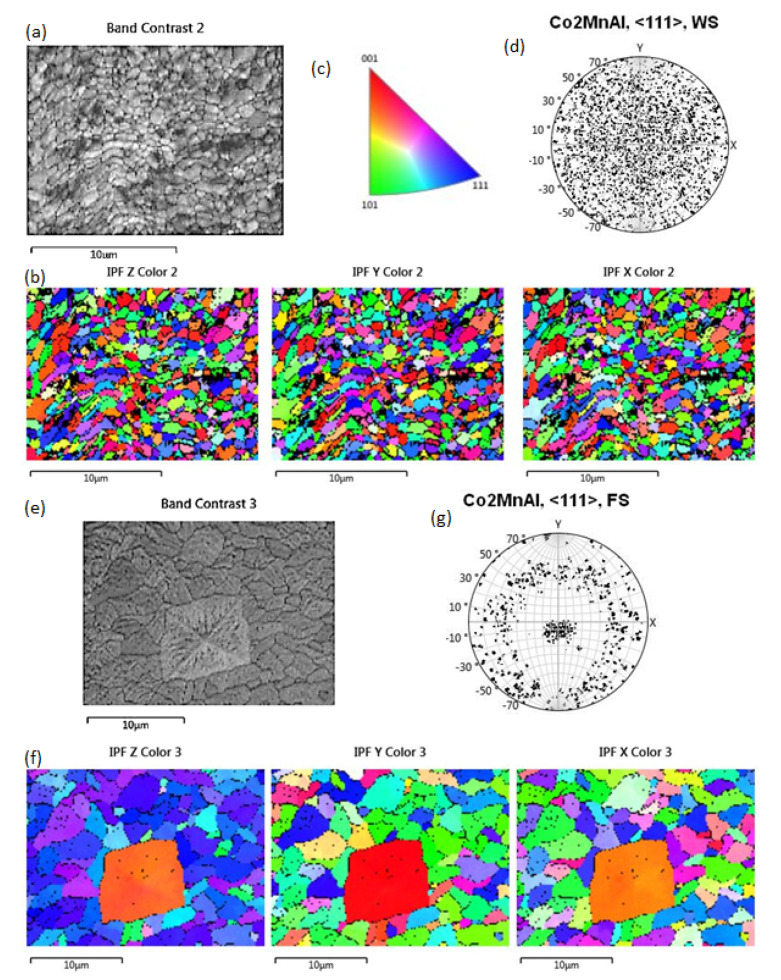
EBSD analysis of the as-spun ribbon surface. At the wheel surface (WS), the crystal orientation of the grains (**a**) is random (**b**,**c**) in all basic directions: perpendicular to the wheel surface -z; transverzal to the wheel rotation -y; parallel to the wheel rotation -x. This is seen also on the pole figure (**d**) constructed from the EBSD signal. Most of the grains at the ribbon free surface (FS) (**e**) have a <111> crystal axis nearly perpendicular to the ribbon surface (**f**). The declination of the <111> axis by about 10 degrees in the y-direction can be seen on the pole figure (**g**). The big square-shaped grain (**f**) nucleated later at the free surface of the ribbon has a different surface morphology and orientation from the rest of the grains.

EBSD analyses performed on the as-grown surfaces are presented in Figure 2. On the wheel side, the fine structure shows a mixture of equiaxed grains and grain elongation in a direction perpendicular to the rotation of the wheel (Figure 2a,b). The linear grain size is 1.1 µm. The crystal orientation of the grains is random in all basic directions (Figure 2b–d). The linear grain size on the free surface of the ribbon is 6 µm (Figure 2e). Most grains have their <111> crystal axis nearly perpendicular to the ribbon surface (Figure 2f). The declination of the <111> axis by about 10 degrees in the y-direction can be very well seen on the pole figure (Figure 2g).

**Figure 3:**
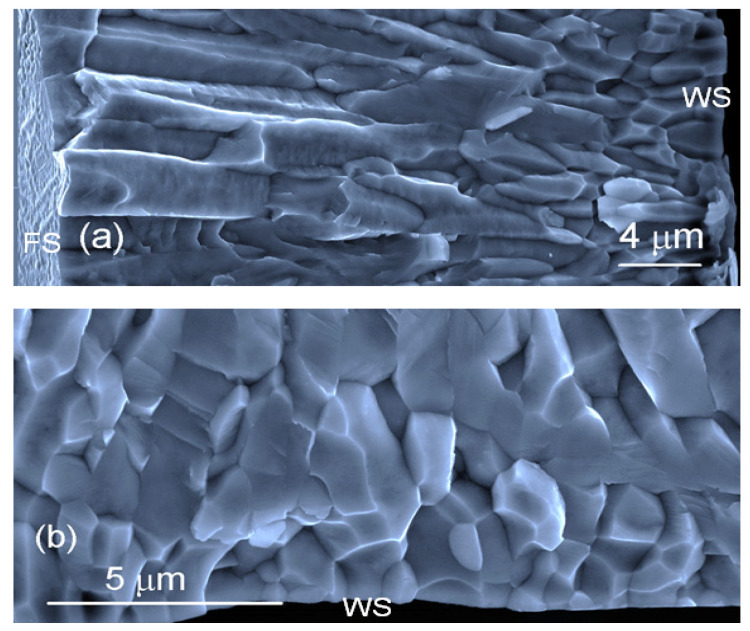
SEM image of the fracture surface of the ribbon. (**a**) The long columnar crystals grow from the wheel side (WS) of the ribbon towards its free surface (FS). (**b**) The small grains developed at the wheel side of the ribbon.

**Figure 4:**
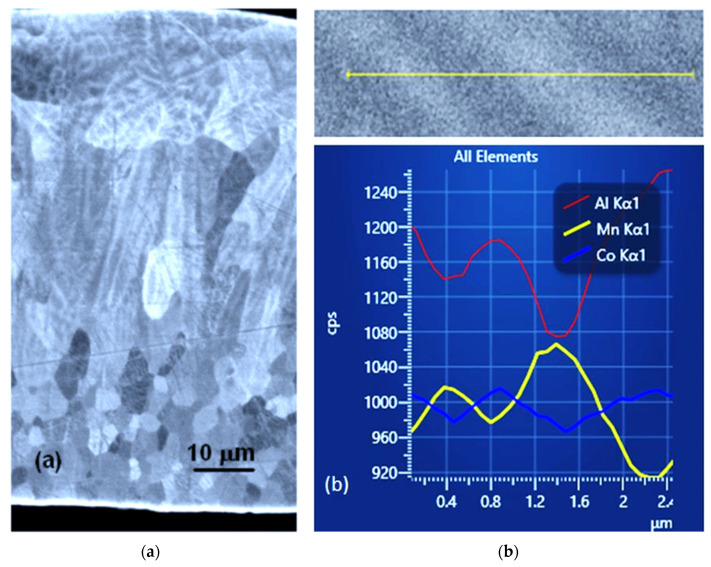
(**a**) Observation in the BSE regime of the ribbon cross-section revealed small grains at the ribbon surface in contact with the wheel, columnar crystals grown by cellular or dendritic growth with darker cell centres, and randomly oriented big grains grown by dendritic growth located at the ribbon free surface. (**b**) EDS line analysis through growth lines and records across the growth lines (cps: counts per second) in columnar grains show that the darker parts are enriched in Co and Al and the brighter parts are enriched in Mn.

The studied ribbons can be easily fractured by bending. On the bending fracture of the ribbon (Figure 3a), we can see that the long columnar crystals grow from the wheel side of the ribbon towards its free surface. They start from small grains developed at the wheel side of the ribbon (Figure 3b). Smaller grains formed on the wheel side of the ribbon can also be seen on the polished cross-section of the ribbon when observed under the BSE. (Figure 4a). Columnar crystals that are more or less perpendicular to the surface of the wheel are developed in the second part of the ribbon. The growth lines in the columnar grains indicate cellular growth. Some randomly oriented grains with dendritic structures may be seen at the free surface of the ribbon. These were obviously nucleated in the melt and do not have any relation to the columnar crystals. The core of the dendrites or cells is darker and the space between them is brighter, suggesting changes in the chemical composition. The EDS line analysis records (Figure 4b) clearly show that the chemical composition of the part of the melt-spun ribbon grown by cellular or dendritic growth is inhomogenous on the micrometer scale (Figure 4b). The darker parts of the growth structure are enriched with Al and Co, while the brighter parts are enriched with Mn. This analysis is consistent with the contrast observed in the BSE mode. BSE signals are stronger if derived from elements with a higher atomic number (atomic numbers Al-13, Mn-25, Co-27).

## 4. Discussion

In melt spinning, the layer of melt that is in contact with the cold wheel is deeply undercooled [16,17]. This allows rapid homogeneous nucleation and the rapid growth of small equiaxed grains. These grains have a random crystal orientation (Figure 2a–d). In places where there is poor contact with the wheel, the undercooling of the melt is not deep enough for the grains to nucleate; therefore, grain growth occurs parallel to the surface of the ribbon (grain elongation in a direction perpendicular to the rotation of the wheel) from areas that have good contact with the wheel (Figure 2b) [18]. Further growth is controlled by the transfer of heat through the solid to the wheel. Grains with a suitable crystal orientation in terms of heat transfer and crystal surface energy grow faster and form dominant columnar crystals (Figure 3a). According to the EBSD analysis, these columnar crystals are parallel to the <111> crystal direction and decline by about 10° against the direction of rotation (Figure 2e,f).

**Figure 5:**
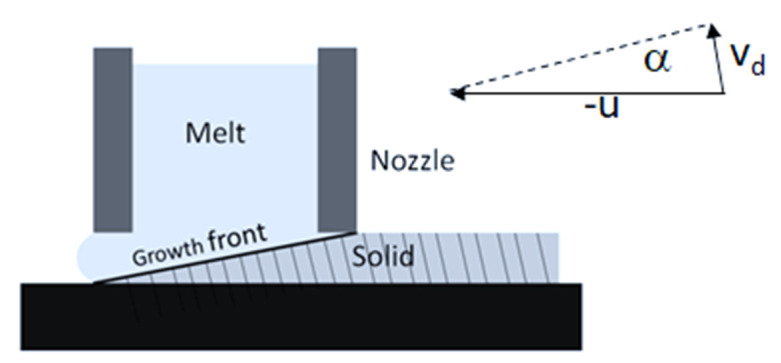
Schematic illustration of the growth front and the related declination of columnar crystals in melt spinning (u: wheel surface speed; v_d_: dendritic growth velocity; α = inclination of columnar dendritic crystals).

This differs from the (100) texture found in the melt-spun ribbons of Ni_2_MnGa-or Ni2MnSn-type Heusler alloys [8,19]. The observed declination has not been reported for the Co_2_MnAl ribbon but is not an uncommon phenomenon in melt-spun ribbons [20,21]. It can be related to the slope of the crystal/melt interface during solidification [22] (Figure 5). Since this slope is the result of the balance between the surface velocity of the wheel *u* and the growth rate of the column crystals *v_d_*, it can be used to estimate *v_d_*. For geometric reasons, the growth rate of a column crystal is equal to *v_d_ = u. sinα.* For the wheel speed *u* = 20 ms^−1^ and the measured declination of the column crystals by 10°, the growth rate is *v_d_* = 3.5 m/s. The estimated growth rate is close to the limit for partitionless solidification [23]. This growth rate limit, *v_dl_*, can be estimated as:

*v_dl_* = *D_i_/δ_i_*, (1)

where *D_i_* is the interfacial diffusion coefficient and *δ_i_* is the atomic jump distance. Using the typical values of *D_i_* = 2.5 × 10^−9^ m^2^/s and *δ_i_* = 0.5 × 10^−9^ m [23], the critical velocity for partitionless growth is calculated to be 5 m/s.

We can assume that the growth rate at the wheel surface of the ribbon was higher than *v_dl_*; therefore, this part of the ribbon was formed by partitionless growth, resulting in the solidification of homogenous Heusler alloy grains without growth lines observed in the columnar crystals (Figure 4a).

The growth structure developed inside the columnar crystals in the form of growth lines is apparently the result of cellular or cellular-dendritic growth [24]. With an increase in the distance from the wheel, the growth rate decreases [13], and when the limit for the partitioning of the Heusler alloy, *v_dl_*, is reached, a contrast appears in the back-scattered electron mode due to the partitioning of Co, Mn, and Al (Figure 4a,b). The X-ray diffraction analysis shows that the sample is a single-phase disordered Heusler alloy. This means that the segregation of the elements during the solidification does not lead to the formation of a new phase. The occupation of the crystal lattice positions with Co, Mn, and Al atoms depends on the position in the grown cell or dendrite, and this nonhomogeneous Heusler alloy should satisfy the following formula: Co_x±a_Mn_y±b_Al_z±c_ with b = a + c.

## 5. Conclusions

In summary, the microstructures of melt-spun Co_2_MnAl samples were investigated. The samples were shown to have typical microstructures associated with crystallization, with fine-grained structures on the wheel side followed by columnar crystals. The columnar crystals grow in the <111> crystal direction and are declined by about 10° against the direction of the spinning, reflecting the slope of the crystal/melt interface during solidification. The estimated growth rate of the columnar crystals is close to but lower than the limit for partitionless crystal growth. Therefore, partitionless growth occurs only at the wheel surface, and partitioned cellular or dendritic crystal growth is observed at the free surface side of the melt-spun ribbon. Therefore, the melt-spun ribbon exhibits a disordered homogeneous Heusler alloy at the wheel side of the ribbon and an inhomogeneous disordered single-phase alloy formed by cellular or dendritic growth at the free surface of the ribbon. The cellular and dendritic growth causes inhomogeneity in the chemical composition, with higher Co and Al concentrations in the centre of the cells or dendrites and a higher concentration of Mn at the cell or dendrite arm boundaries.

## Data Availability

Data sharing not applicable.
